# Synergistic Effects of Inflammation and Drug Interactions on CYP3A5*3/*3 Phenoconversion in Antipsychotic Metabolism

**DOI:** 10.3390/pharmaceutics18070782

**Published:** 2026-06-26

**Authors:** Krisztina Kőhalmy, Ayaan Borthakur, Pálma Porrogi

**Affiliations:** 1 Independent Researcher, 2040 Budaörs, Hungary; kohalmy.krisztina@gmail.com; 2 Independent Researcher, Guwahati 781028, India; ayaanborthakur2019@gmail.com; 3 Faculty of Health Sciences, Széchenyi István University, 9026 Győr, Hungary

**Keywords:** phenoconversion, pharmacogenetics (PGx), drug–drug interaction (DDI), inflammation, genotype-phenotype gap, quetiapin

## Abstract

**Background:** Traditional genotype-guided dosing often fails to predict real-time variability in the metabolic phenotype during complex polypharmacy. This secondary analysis of a retrospective cohort aims to elucidate mechanisms underlying real-time phenoconversion during antipsychotic therapy, focusing on homozygous loss-of-function CYP3A5*3/*3 non-expressors. **Methods:** Using an additive phenoconversion model that integrates a genotype-derived baseline with environmental modifiers for drug–drug interactions (DDI), systemic inflammation (CRP), and renal function (eGFR), we demonstrate that the expressed metabolic phenotype is a dynamic, context-dependent construct that can markedly diverge from the genotype-predicted state. **Objectives:** Our data show that patients with CYP3A5*3/*3 and CYP3A inhibitors (e.g., ritonavir) had a quetiapine plasma concentrations reached 1850 ng/mL, corresponding to 3.7-fold above the internationally accepted therapeutic reference range of 100–500 ng/mL. Acute systemic inflammation (CRP > 50 mg/L) induced a functional poor metabolizer phenotype (P*_act_* < −0.9) in individuals with a genotypic normal metabolizer status. In contrast, strong inducers such as carbamazepine, phenytoin, and heavy smoking promoted an ultra-rapid metabolizer state (CL_ind_ > 4.0 L/h, quetiapine < 30 ng/mL), consistent with treatment failure. In this cohort, the additive P*_act_* model showed a strong association with observed clearance and identified clinically relevant phenoconversion mechanisms not predicted from genotype alone. **Conclusions:** These results support a dynamic, multi-parametric approach that integrates pharmacogenomics, therapeutic drug monitoring, biomarker profiling (CRP, eGFR), and structured DDI assessment to enable higher-resolution, real-time phenotype tracking and more informed dose individualization in high-risk psychiatric polypharmacy.

## 1. Introduction

Despite the rapid integration of pharmacogenomics into clinical practice, the ‘genotype-to-phenotype’ gap remains a significant barrier to patient safety. Pharmacogenomic (PGx) testing identifies genetic variants in drug-metabolizing enzymes and is increasingly used to guide dose selection and reduce adverse effects [1]. The CYP3A4 and CYP3A5 enzymes are central to the metabolism of antipsychotics such as quetiapine, risperidone, and aripiprazole. Genotyping for loss-of-function alleles such as CYP3A5*3 (rs776746) identifies individuals at higher risk for reduced drug clearance and toxicity [2,3]. However, genotype alone often fails to predict the actual metabolic phenotype because factors such as phenoconversion can cause metabolic capacity to differ from genotype-based expectations [1,4,5].

This genotype-phenotype discrepancy is significant in antipsychotic polypharmacy and other therapies. Polypharmacy, common in complex psychiatric disorders and necessary in many treatments (e.g., infection, immunosuppression), poses risks of drug–drug interactions (DDIs) that affect drug metabolism, especially when multiple drugs impact the same enzyme pathways [4,6]. Patient factors such as inflammation, renal impairment, obesity, age, and smoking increase the risks of drug accumulation and side effects, risking treatment failure. These challenges, clinical guidelines lag behind emerging scientific multi-omics evidence. They rely mainly on genotypic data or delayed therapeutic drug monitoring (TDM), which do not reflect the patient’s current state, limiting timely intervention [7,8]. Monitoring methods that account for physiological factors include measuring plasma drug concentrations, observing biomarkers such as C-reactive protein (CRP) and IL-6, and reviewing drug regimens to identify high-risk combinations [6,9]. These measures may help detect toxicity or an inadequate response early and inform future guideline updates.

This secondary, supplementary analysis examines four *CYP3A5* patients from a retrospective cohort on antipsychotic polypharmacy. Using pharmacokinetics, biomarkers, and modeling, it shows that rapid phenoconversion results from DDIs, polypharmacy, renal function, and inflammation, not genotype alone. The toxicity risk depends on these combined factors. While focused on CYP3A5*3/*3, the pharmacokinetic and clinical interactions may also apply to other genotypes or patients with polypharmacy, inflammation, or organ impairment. The dynamic, multiparameter monitoring principles suggested may help prevent toxicity in high-risk psychiatric populations.

### 1.1. Phenoconversion at the Molecular Level

At the molecular level, DDIs in psychiatric polypharmacy result in complex disruptions of CYP enzyme networks, fundamentally altering pharmacokinetics through competitive, non-competitive, and mechanism-based inhibition. Competitive inhibition occurs when multiple substrates, such as quetiapine, risperidone, and clonazepam, bind to overlapping ligand-binding sites within the active site of *CYP3A4* (7q21.3–q22.1) or *CYP3A5* [2,3,4,10,11]. This binding causes direct steric hindrance and substrate competition, leading to an increased apparent Michaelis–Menten constant (Km), decreased maximum velocity (v_max_), and non-linear, saturable elimination kinetics [2,12]. Clinically, this is observed as disproportionate increases in plasma drug concentrations when substrate load exceeds enzymatic capacity.

Environmental and systemic physiological factors further modulate CYP gene expression and activity through complex molecular signaling and epigenetic regulation. Pro-inflammatory cytokines, including IL-6, TNF-α, and IL-1β, activate intracellular pathways such as JAK/STAT3 and NF-κB. These pathways converge on the promoters of CYP3A4 and CYP3A5, repressing transcription by recruiting histone deacetylases (HDACs) and DNA methyltransferases (DNMTs) [9,13,14,15]. For instance, CpG island hypermethylation at the CYP3A4 promoter and increased H3K27 trimethylation (H3K27me3) at enhancer regions result in sustained transcriptional silencing during systemic inflammation [16,17]. Although these modifications are reversible, they can persist during inflammation, reducing enzyme activity. Additionally, microRNAs such as miR-27b and miR-148a regulate CYP3A4 expression, adding an additional layer of control [18]. This regulation explains cases where genotypic normal metabolizers (gNM) may phenotypically present as intermediates (fIM) or poor metabolizers (fPM) under epigenetic or inflammatory influences. The effect of DDIs on kinetics varies by substrate and inhibitor. Potent inhibitors such as ritonavir and ketoconazole form covalent adducts with CYP3A4, causing quasi-irreversible enzyme inactivation and reducing v_max_. Competitive inhibitors such as risperidone increase Km without affecting v_max_, lowering catalytic efficiency. During polypharmacy, metabolic crowding at CYP3A4/5 may lead to saturable kinetics and toxic interactions. Enzyme inducers such as carbamazepine and rifampicin activate PXR and CAR, upregulating *CYP3A4* and *CYP2C9* by promoting histone acetyltransferase recruitment and chromatin decondensation [2,19,20,21]. This decreases steady-state drug levels, reducing accumulation but risking subtherapeutic exposure. In antipsychotic polypharmacy, these mechanisms create variable metabolic capacity. Co-administration of multiple CYP3A substrates, especially with strong inhibitors, can saturate and inactivate the enzyme at transcriptional and post-translational levels [2,21]. For example, a patient with a CYP3A5*3/*3 genotype (loss-of-function) exposed to ritonavir will experience compounded genetic and acquired CYP3A loss, dramatically increasing the quetiapine AUC and half-life and decreasing clearance beyond genotype predictions. These molecular events have direct clinical implications. For instance, strong CYP3A inhibition in patients receiving quetiapine can raise the AUC by over 500% and extend the half-life (t½) from 7 to 24 h, increasing the risk of toxicity [22]. Conversely, tobacco smoke induces CYP1A2 via AhR upregulation at locus 15q24.1, lowering olanzapine exposure below therapeutic levels and reducing efficacy (Table 1) [5,23,24].

This analysis emphasizes recognizing the risk in antipsychotic polypharmacy, showing that thorough medication review and monitoring of metabolizer status can improve prediction and prevention of adverse reactions. Linking polypharmacy-induced phenoconversion to failure or toxicity highlights the importance of integrating metabolic assessments into routine practice, enhancing patient safety beyond current genotype-guided methods.

### 1.2. Limitations of Current Guidelines and the Role of Therapeutic Drug Monitoring as a Real-Time Phenotypic Probe

International consortia like DPWG and CPIC offer genotype-based drug dosing recommendations, with AGNP guidelines highlighting TDM alongside PGx [34,35]. However, these frameworks often operate separately and lack protocols for changes over time and for physiological or behavioral influences on phenotypic conversion. For instance, a patient with CYP3A5*3/*3 taking multiple drugs, including a CYP3A inhibitor like ketoconazole, and having acute kidney injury, faces increased risks [25,27]. The inhibitor reduces CYP3A activity, and kidney injury hampers drug elimination, raising toxicity risks beyond genotype predictions. Polypharmacy further worsens this by competing for or inhibiting metabolic pathways. Therefore, despite guidance, such patients can develop unexpected toxicity from combined polypharmacy and organ dysfunction, risks not fully addressed by current guidelines.

### 1.3. Biomarker Integration: The Role of CRP and eGFR in Modulating Metabolic Capacity

CRP signals systemic inflammation, with cytokines like IL-6 suppressing CYP expression and sharply reducing drug clearance, independent of genotype. Low eGFR also heightens risk through uremic inhibition of hepatic CYPs, while hypoalbuminemia alters drug distribution. Combined CRP elevation and renal impairment greatly increase susceptibility to toxic accumulation, especially under enzyme inhibition or metabolic crowding. These complex interactions necessitate quantitative, model-based approaches: dynamic integration of PGx-TDM-biomarker data and kinetic modeling enables real-time reclassification of phenotypes and dosing adjustments [6,7,36,37]. Kinetic and vectorial models are clinically valuable for predicting rapid DDI effects and identifying critical 48–72 h intervention windows to prevent severe toxicity.

To bridge the gap between static guidelines and dynamic clinical reality, this study introduces an additive Phenoconversion Model (P*_act_*). By integrating the genetic anchor (G*_base_*) with additive environmental modifiers for drug–drug interactions, inflammation, and renal function, the model provides a high-resolution, real-time predictive tool for antipsychotic dosing in high-risk psychiatric populations (Equation).

### 1.4. Identifying Knowledge Gaps and Study Rationale

Although the individual contributions of PGx, DDIs, inflammation, and renal impairment are well documented, there is a critical lack of integrated clinical analyses showing how these factors together cause rapid and severe phenoconversion in psychiatric patients. This supplementary analysis of a retrospective cohort aims to address this translational gap. There is a critical systemic lack of integrated frameworks that capture their non-linear convergence. Current clinical practice largely relies on static genetic markers, which often fail to reflect the patient’s dynamic metabolic state. This discrepancy highlights a fundamental translational gap: genotype is an inherited static blueprint, but it is not metabolic destiny. In complex psychiatric populations, the cumulative pressure of environmental and physiological stressors can effectively override genetic predispositions through a process of rapid and severe phenoconversion. This article addresses this gap by analyzing four paradigmatic cases in which the ontological shift from a predicted to a functional phenotype was clinically observed. By integrating detailed molecular profiling with our proposed additive phenoconversion model, we demonstrate that genetic susceptibility (CYP3A5*3/*3), metabolic congestion, and inflammatory suppression synergistically create a state of ‘metabolic collapse. These cases provide empirical validation for the hypothesis that real-time, model-based monitoring is not merely an adjunct but a necessity to prevent life-threatening drug accumulation when static PGx guidelines reach their predictive limits.

## 2. Methods

### 2.1. Data Collection; Study Design

This study was designed as a secondary, supplementary analysis of a well-characterized retrospective cohort derived from a highly controlled research program focused on pharmacokinetic risks in psychiatric pharmacotherapy. Transitioning from a descriptive case series framework, this approach evaluates 24 patients diagnosed with major psychiatric disorders such as schizophrenia, schizoaffective disorder, or bipolar disorder. Patients were included if they were receiving either: (1) antipsychotic polypharmacy, defined as the concomitant use of two or more antipsychotics for at least 7 days, or (2) antipsychotic monotherapy (e.g., quetiapine or risperidone alone. The monotherapy patients were strictly defined as a separate, matched pharmacokinetic internal reference (control) group, to which the main polypharmacy criterion did not apply. This reference group serves to establish baseline pharmacokinetic profiles, enabling a clearer evaluation of potential DDI in the polypharmacy cohort. Exclusion criteria were incomplete clinical or laboratory records, genotyping failure, or the presence of acute, life-threatening comorbidities (e.g., acute hepatic failure) that could independently and significantly influence drug metabolism and confound the analysis of DDI-driven phenoconversion. This secondary analysis of a retrospective cohort used data from a highly controlled research program on pharmacokinetic risks in psychiatric pharmacotherapy. Due to confidentiality, specific details like institution name and location cannot be disclosed. The study was approved by the Hungarian Committee of Science and Ethics, Medical Research Council. It was performed under the regulations of Act CLIV of 1997 on Health and the decree 23/2002 of the Minister of Health of Hungary, and in accordance with the Declaration of Helsinki [38], the European Union General Data Protection Regulation (GDPR). All patients belonged to the Caucasian population, and their demographic and clinical data as well as the details of pharmacotherapy (daily dose, serum concentration) were recorded (Supplementary Materials).

### 2.2. Genotyping

Genotyping was performed using validated PCR-based assays to determine CYP variants. Amplification was performed using a CFX Opus Real-Time PCR System (Bio-Rad, Hercules, CA, USA) with reagents supplied by Bio-Rad Magyarország Kft. (Budapest, Hungary).Genomic DNA was extracted from EDTA-stabilized peripheral blood using the High Pure Template Preparation Kit (Roche Diagnostics GmbH, Mannheim, Germany) and quantified using a NanoDrop 1000 (Thermo Fisher Scientific, Wilmington, DE, USA) [39,40]. Genotyping of *CYP3A4* (*1/*1, *1/*22) and *CYP3A5* (*1/*1, *1/*3) was performed with allele-specific TaqMan probes (LGC Biosearch Technologies, Petaluma, CA, USA) on a real-time PCR platform [41,42]. Patients were categorized as either CYP3A5 *3/*3 (homozygous loss-of-function) or normal metabolizers (gNM; CYP3A4 *1/*1, *1/*22 and CYP3A5 *1/*1, *1/*3). The reference group comprised patients with wild-type *CYP3A4* and *CYP3A5* genotype, no exposure to strong CYP3A inhibitors, and preserved renal function (eGFR > 70 mL/min/1.73 m^2^).

### 2.3. Pharmacokinetic Analysis

Serum concentrations were determined using a validated LC-MS/MS method (Perkin Elmer Series 200 micro LC (PerkinElmer, Inc., Waltham, MA, USA) coupled to an AB Sciex 3200 QTRAP mass spectrometer (AB Sciex, Framingham, MA, USA)). After methanol protein precipitation, detection was performed in positive ESI MS/MS (MRM) mode [8,43]. Methyl-risperidone served as the internal standard. Separation was achieved on a Zorbax SB C1 column (Agilent Technologies, Santa Clara, CA, USA) with a water-acetonitrile gradient (formic acid/ammonium formate (Sigma-Aldrich, St. Louis, MO, USA)).

Phenoconversion was quantitatively assessed using a semi-quantitative scoring system that was developed to integrate multiple metabolic modifiers. The dynamic phenotypic activity score (P*_act_*) quantifies real-time divergence between genetic metabolic potential and the observed functional phenotype. P*_act_* integrates constitutional genotype and principal environmental modifiers within a vectorial phenoconversion framework, thereby capturing the dynamic nature of drug metabolism.

With no validated weights available for CYP3A5-based phenoconversion, the P*_act_* score was formulated based on a reference-based additive model.

P*_act_* is constructed as a linear, additively scaled composite score rather than a multiplicative penalty to improve interpretability, internal consistency, and clinical applicability. Genotype is represented as a categorical baseline descriptor (G_base_), assigning no penalty for gNM and gIM and a fixed negative shift only for gPM (G_base_ = 0.00 for gNM and gIM; −0.50 for gPM). This approach reflects the established loss-of-function of poor metabolizers and avoids unsupported numerical penalties for intermediate phenotypes, consistent with current pharmacogenetic guideline practices (e.g., CPIC, DPWG). Environmental and clinical modifiers, including drug–drug interactions (Δ_DDI_), inflammation (Δ_Inf_), and renal function (Δ_Ren_), are incorporated as additive, prespecified stepwise penalties or bonuses to the P*_act_* score:

P*_act_* = G_base_ + Δ_DDI_ + Δ_Inf_ + Δ_Ren_ (1)

where G_base_ is the genotype-based susceptibility, Δ_DDI_ corresponds to the impact of DDI, Δ_Inf_ denotes inflammation-induced suppression based on CRP, and Δ_Ren_ corresponds to renal impairment according to eGFR.

In this framework, Δ_DDI_, Δ_Inf_, and Δ_Ren_ are defined in discrete, biologically and clinically motivated strata: none, mild, moderate, or strong inhibition or induction for DDI; CRP bands for inflammation; and eGFR categories for renal function. Each modifier has a bounded effect size (e.g., up to −0.25 for strong inhibition and +0.50 for strong induction, up to −0.80 for severe inflammation) (Supplementary Materials).

Modification factors were obtained using clinical cut-offs and pharmacokinetics interaction categories. The modification factors were chosen in an empirical way to keep the concordant genotype-phenotype reference group at P*_act_* = 0 and reproduce the phenotypic hierarchy as it was observed in patients. The levels of drug concentrations and individual clearances were not part of the P*_act_* determination process but represented the pharmacokinetic endpoint variables separately.

### 2.4. Statistical Analysis

Correlations were assessed using Pearson’s or Spearman’s correlation coefficients. Statistical significance was set at *p* < 0.05. All analyses were conducted using GraphPad Prism v10 and R v4.3. (Version 10.0.0, GraphPad Software, Boston, MA, USA).

## 3. Results

This secundary analysis aims to elucidate the molecular and pharmacokinetic mechanisms underlying phenoconversion in a well-defined patient population (*n* = 24) exposed to complex psychiatric polypharmacy in real-world settings. The study quantitatively shows that non-genetic factors, especially chronic inflammation, measured by CRP as a biomarker) and CYP3A-mediated DDI, significantly alter drug kinetics. These changes may cause clinically significant, potentially life-threatening drug accumulation and toxicity, even in patients whose genotype alone would not predict such risk.

The results indicate that the metabolic phenotype is not a direct, static but a dynamically modifiable and variable manifestation shaped by the combined, and sometimes synergistic, effects of the inherited genotype, pharmacological exposure, physiological state, and environmental factors. Three principal conclusions are drawn from this study:

A stable, genotype–phenotype concordant reference subgroup (gNM and fNM), within which a stringently defined, drug- and inflammation-free monotherapeutic subset functions as an internal absolute control, provides the pharmacokinetic baseline against which phenoconverted cases are quantitatively compared. The CYP3A5*3/*3 genotype confers a marked predisposition, with its clinical manifestation being decisively determined by the pharmacological environment, such as inhibition and metabolic crowding, which can result in extreme drug accumulation.

Chronic inflammation, indicated by elevated CRP, alone can convert even wild-type genotype patients into a fully fPM state, thereby completely neutralizing the predictive value of genotype [1,4,24].

### 3.1. Clinical Reference Definition: The Genotype-Phenotype Concordance Cases

To establish a physiological baseline for CYP3A5-mediated metabolism, we defined a genotype–phenotype concordant reference subgroup comprising six patients (P3, P8, P11, P14, P19, P23), selected based on inclusion and exclusion criteria specified in Section 2.1 a rigorous, multiconsistent review of systematic electronic health record data. Within this reference subgroup, two monotherapy patients (P3 and P14), free of relevant inflammation, toxicity, and interacting co-medications, were further designated as an internal pharmacokinetic control subset, providing the primary pharmacokinetic baseline for all comparative analyses (Table 2). The 30-day medication history prior to clinical sampling in the study was extracted from each patient’s clinical records to identify pharmacones and DDIs involving CYP3A modulators or other potential drug–drug interactions. Laboratory values, including liver and kidney function markers and CRP levels, were reviewed to confirm the absence of significant organ damage or inflammation at baseline. Inclusion required at least one functional CYP3A5*1 allele (gNM), and the absence of significant liver or kidney damage. Subjects with drug interactions affecting CYP3A enzymes, significant systemic inflammation (CRP >3 mg/L), or use of strong enzyme inducers or inhibitors within 30 days prior to the study were excluded. These patients demonstrated stable pharmacokinetics without significant confounding factors. Their mean CL_ind_ was 2.12 ± 0.15 L/h. By day 8 of therapy, plasma concentrations of quetiapine and risperidone remained within the established therapeutic windows (e.g., quetiapine: 95–130 ng/mL). This reflects minimal drug interactions and low systemic inflammation (mean CRP < 3 mg/L). Thus, this concordant case serves as a metabolic reference for assessing the effects of phenoconversion.

This tabular framework illustrates the intersection between genetic vulnerability (CYP3A5 expression status) and exogenous kinetic stressors—such as polypharmacy, inflammation, inhibition, or induction. They drive real-time phenoconversion within the evaluated patient population. To ensure methodological transparency, the specific distribution of the 24 subjects derived from the retrospective parent cohort is explicitly mapped across the established groups. Subgroup A.1 (*n* = 2) comprises patients P3 and P14, serving as internal pharmacokinetic control subset, while Subgroup A.2 (*n* = 4) includes patients P8, P11, P19, and P23, representing polypharmacy with a minimal DD) burden; together, these subgroups constitute Group A as the concordant, Genotypic Normal Baseline reference exhibiting stable steady-state kinetics (Table 2). Group B (*n* = 8) consists of patients P1, P4, P6, P12, P15, P17, P21, and P24, isolating individuals with Genotypic Poor status under Metabolic Crowding driven by multi-substrate competition. Group C (*n* = 2) includes patients P7 and P13, representing Genotypic Normal individuals undergoing Inflammatory Phenoconversion due to severe cytokine-driven enzymatic suppression. Group D (*n* = 2) is formed by patients P9 and P18, capturing Genotypic Poor individuals exposed to Critical Inhibition. Lastly, Group E (*n* = 6) encompasses patients P2, P5, P10, P16, P20, and P22, categorizing Genotypic Normal individuals undergoing Inducer-Driven Phenoconversion via nuclear receptor-mediated transcriptional upregulation.

The genetically deficient CYP3A5*3/*3 homozygous non-expressor individuals (rs776746:*AA*)—are explicitly highlighted in the main manuscript table with a light gray background shading.

### 3.2. Metabolic Risk Effects of the CYP3A5*3/*3 Genotype

The homozygous CYP3A5*3/*3 genotype (gPM) carries a significant genetic risk for reduced drug clearance, as it is associated with reduced CYP3A5 enzyme activity [41,44,45]. However, the clinical effect of this enzyme genotype is highly dependent on the pharmacological environment, which may result in two major risks. In CYP3A5-deficient individuals, metabolic capacity is almost entirely dependent on residual CYP3A4 function, which has been shown to compensate for CYP3A5 deficiency to a variable extent [46]. Additional factors, such as competing substrates, inhibitors, or inducers, can significantly alter CYP3A4 enzyme activity, either reducing clearance and thereby causing drug accumulation and leading to subtherapeutic levels. The interaction between genotype and pharmacological microenvironment ultimately determines the metabolic capacity and metabolic risk of individual patients. For example, in the current cohort, patient P1 with the CYP3A5*3/*3 genotype had significantly increased quetiapine concentrations when exposed to multiple CYP3A substrates, compared with patients with pre-existing reduced CYP3A4 activity, highlighting that genetic deficiency combined with specific pharmacological factors can significantly impair drug clearance. These results demonstrate the need to consider both genetic background and current pharmacological factors when prescribing drugs metabolized by CYP3A, and emphasize the importance of individual, real-time monitoring to minimize side effects and optimize therapeutic outcomes.

In patients P1, P6, and P21, coadministration of several highly lipophilic CYP3A4 substrates, including quetiapine, risperidone, clonazepam, and a lipophilic statin, caused significant competitive saturation of the residual CYP3A4-mediated metabolic capacity. This phenomenon, called enzymatic CYP3A4 crowding, occurs because these drugs compete simultaneously for the same active catalytic sites of the isoenzyme, severely limiting the biotransformation rate of each agent. Quantitatively, this enzymatic bottleneck appeared as marked pharmacokinetic nonlinearity. Metabolic pathways became saturated, leading to a sharp, disproportionate increase in quetiapine plasma concentrations by day 4, peaking at 1850 ng/mL in patient P1. To rigorously interpret overexposure, these values were evaluated relative to both international clinical standards and the internal cohort baseline. The internationally recognized therapeutic reference range, defined by the AGNP Consensus Guidelines, specifies a therapeutic window of 100 to 500 ng/mL for quetiapine to minimize toxicity and maximize efficacy. The peak concentration in patient P1 was 3.7 times above this range, placing it in a highly supratherapeutic and dangerous zone. To further clarify the magnitude of this interaction, exposure was also assessed relative to an internal retrospective cohort-derived reference group (Group A, representing monotherapy and non-interacting polydrug regimens). In Group A, the baseline steady-state concentration remained stable at 96.17 ± 20.35 ng/mL, indicating that, without enzymatic competition, quetiapine pharmacokinetics are predictable and remain within the lower limits of the therapeutic window. Consequently, patient P1 showed a 19.2-fold increase in concentration above the internal cohort baseline mean. The main cause of systemic accumulation was the mean CL_ind_ of patients P1, P6, and P21, which decreased to 0.53 ± 0.06 L/h and represented only 25% of the mean clearance capacity in the internal cohort reference group (2.12 ± 0.15 L/h; *p* < 0.001). This reduction in metabolic clearance capacity directly explains the observed nonlinear drug accumulation, as the body’s ability to eliminate the drug was insufficient to compensate for the daily dosing. Ultimately, this critically increased individual pharmacokinetic burden results in an accelerated clinical risk of serious, dose-dependent adverse drug reactions.

**Figure 1:**
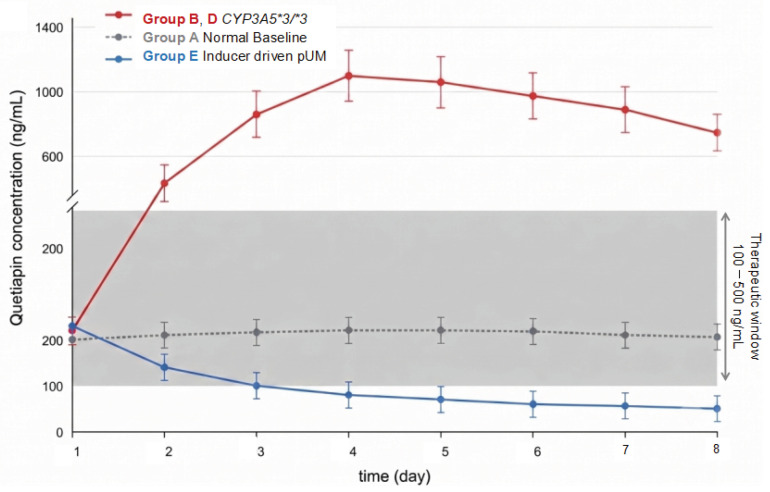
Longitudinal quetiapine plasma levels across different metabolic phenotypes from Day 1–8.

The most severe outcomes were observed in patients P9 and P18, where the genetic deficiency was combined with strong CYP3A inhibitors (ritonavir and cobicistat). This combination resulted in a critical inhibition and accumulation phenotype, with CL_ind_ reduced to 0.5 L/h and quetiapine concentrations remaining above 1000 ng/mL on day 8 (Figure 1). This indicates a complete loss of compensatory metabolic pathways.

The high inter-individual variability and the rapid onset of critical accumulation in phenoconverted patients suggest that proactive TDM on day 4 is a vital safety intervention for patients presenting with elevated inflammatory markers or complex polypharmacy.

Data points indicate sub-cohort means from the retrospective validation cohort, with vertical error bars showing standard deviation (±SD). The *Y*-axis uses a structural scale break to visualize sub-therapeutic and baseline pharmacokinetic ranges clearly, while also capturing substantial non-linear drug accumulation in the toxic zone. The dark shaded region marks the consensus therapeutic window for quetiapine (100–500 ng/mL) as established by the AGNP guidelines.

The red profile represents Groups B and D, which consist exclusively of genetically vulnerable CYP3A5*3/*3 homozygous non-expressors. In this sub-cohort, the lack of functional CYP3A5-mediated compensatory clearance combined with intensive exogenous kinetic stressors such as multi-substrate competitive CYP3A4 crowding from co-administered lipophilic agents (quetiapine, risperidone, clonazepam, and a lipophilic statin) or mechanism-based inhibition results in profound metabolic collapse. This enzymatic bottleneck causes a sharp, highly nonlinear pharmacokinetic escalation, with group-mean plasma levels exceeding the upper therapeutic ceiling within 48 h and peaking at day 4 (~1100 ng/mL). This phenoconversion is due to extreme individual accumulation, as shown by patient P1, who exhibited a 3.7-fold systemic overexposure above the AGNP therapeutic limit and a 19.2-fold increase above the internal baseline mean (96.17 ± 20.35 ng/mL). In contrast, Group A (Grey, Normal Baseline), which includes both monotherapy and non-interacting polypharmacy regimens, maintains stable steady-state plasma concentrations throughout the 8-day monitoring period. These findings confirm that quetiapine pharmacokinetics remain predictable and within the lower bounds of the therapeutic window when enzymatic competition is absent. Group E (Blue, Inducer-driven fUM) demonstrates rapid transcriptional phenoconversion. Nuclear receptor activation (PXR/AhR) by potent xenobiotic inducers such as carbamazepine or heavy tobacco smoking, accelerates quetiapine clearance. As a result, mean plasma levels fall below the minimum therapeutic threshold (~100 ng/mL) by day 2 and drop below 50 ng/mL by day 4, increasing the risk of therapeutic failure.

Each point represents an individual patient, grouped by genotype and environmental modifiers. Group means highlight distinct metabolic states: concordant baseline (A, mean 0.00), metabolic crowding (B, mean −0.82), inflammatory phenoconversion (C, mean −0.62), critical inhibition (D, mean −0.97), and inducer-driven phenoconversion (E, mean +0.50). Patient-level distribution of P*_act_* scores across phenoconversion groups. Horizontal dashed line at Pact = 0 represents the concordant reference value of the genotype–phenotype relationship, while the dotted red line at Pact = −0.90 depicts the threshold value indicating the critical level of metabolic dysfunction. Boxes in the boxplot demonstrate the interquartile range (IQR), and the median and mean values are shown as solid black and dashed lines, respectively. If Pact is negative, it means that the functional level of metabolic activity is decreased; if positive, then the increased metabolic activity is associated with induction.

### 3.3. Cytokine- and DDI-Driven Phenoconversion

**Figure 2:**
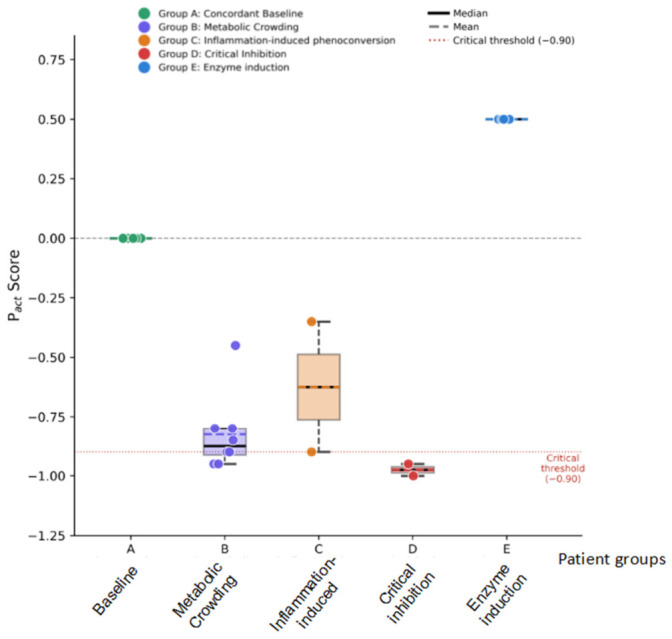
Patient-level distribution of P*_act_* scores across phenoconversion groups.

**Figure 3:**
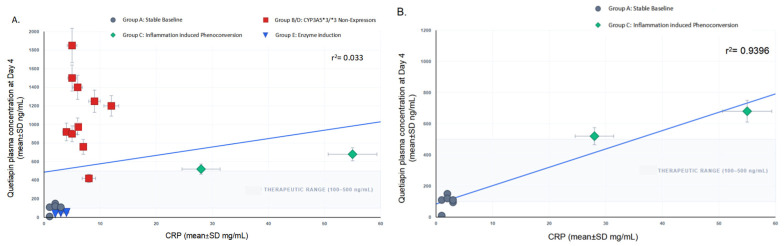
Distinct genetic and inflammation-mediated mechanisms underlie CYP3A phenoconversion and quetiapine accumulation. (**A**) Linear regression analysis of quetiapine plasma concentration at Day 4 versus CRP levels in the entire patient cohort (*n* = 24, r^2^ = 0.033) shows no significant global correlation, indicating mechanistic heterogeneity. Red squares identify clinical outliers with the CYP3A5*3/*3 genotype and predicted poor metabolizer (fPM) activity who show marked drug accumulation independent of systemic inflammation. Green diamonds indicate the inflammation-driven phenoconversion subgroup. Blue triangles represent inducer-driven patients with subtherapeutic drug concentrations clustered near the origin. Grey circles correspond to baseline patients who maintain metabolic stability within or near the therapeutic range (shaded area, 100–500 ng/mL). (**B**) Targeted linear regression comparing the inflammation-driven subgroup to the baseline reference (*n* = 6) demonstrates a near-deterministic relationship (r^2^ = 0.9396). This strong association establishes CRP as a precise clinical surrogate for cytokine-mediated CYP3A4 suppression and the resulting exposure risks. Error bars indicate mean ± SD.

**Figure 4:**
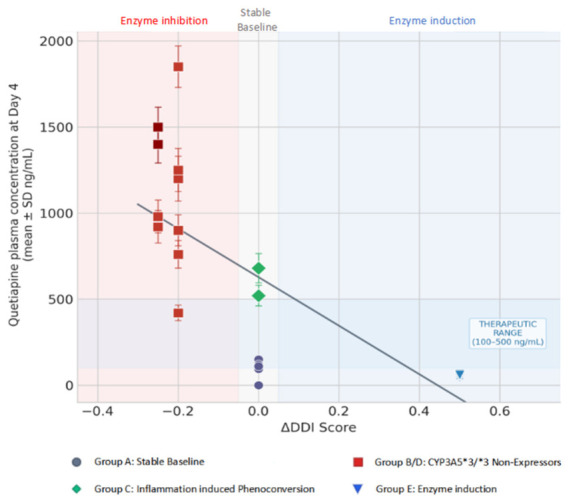
Inverse relationship between metabolic DDI potential and quetiapine systemic exposure across distinct clinical phenotypes.

Systemic and acute inflammation alone can cause severe phenoconversion, overriding the profile predicted by the genetic allele. Patient P7, with the wild-type CYP3A5*1/*1 gNM, displayed a fPM phenotype with a CL_ind_ of 0.7 L/h and a P*_act_* score of −0.94 (Figure 2). This metabolic decline was directly related to the patient’s acute inflammation (CRP: 55 mg/L). The process of phenotypic shift involves cytokine-mediated suppression of CYP gene expression, particularly through IL-6, IL-1β and nuclear receptor pathways (e.g., PXR/RXR). In summary, pro-inflammatory cytokines, especially IL-6, inhibit the transcription and translation of CYP3A4 and CYP3A5, leading to decreased metabolic enzyme activity and impaired drug clearance (Figure 3 and Figure 4).

The rapid, non-linear drug accumulation observed across patient cohorts highlights two clinically significant but mechanistically independent pathways of CYP3A4/5 enzyme suppression. In Group C (Inflammatory Phenoconversion), demonstrated by patients P7 and P13, pharmacokinetic deviation is primarily attributable to systemic inflammation. Elevated CRP levels, averaging 55 mg/L in P7 and 28 mg/L in P13, serve as clinical markers for an underlying pro-inflammatory cytokine cascade involving IL-6, IL-β, and TNF-α. These cytokines are hypothesized to mediate transcriptional down-regulation of the *CYP3A4* gene, most likely via nuclear receptor cross-talk—potentially through interference with the PXR/RXR heterodimer binding to the promoter region—although direct mechanistic evidence from patient-derived measurements is lacking. This putative environment-driven enzyme suppression is associated with phenotypic discordance, in which gNM converts to fPM. The resulting reduction in intrinsic clearance (CL_Ind_ reduced to 0.7 L/h in P7) leads to a rapid increase in quetiapine concentrations to supratherapeutic levels (680 ng/mL by Day 4) (Figure 3A,B).

The inflammation-driven exposure trajectory was validated using a targeted regression model that isolated cytokine-driven individuals and baseline controls, resulting in a near-deterministic linear fit (r^2^ = 0.9396) (Figure 3B). Due to the exploratory design and the lack of an independent validation dataset, we performed robust bootstrapping analyses (1000 resamplings) to generate stable 95% confidence intervals (CIs) for the slope, reducing the risk of overfitting due to the small sample size (n = 6). The association shows that, in the presence of systemic inflammation, CRP levels are closely correlated with the severity of phenoconversion. However, formal predictive efficacy, including sensitivity and specificity thresholds, requires validation in larger, external cohorts. This correlation is absent in the unstratified total population (r^2^ = 0.033; Figure 3A), where severe quetiapine accumulation is driven by a distinct, non-inflammatory mechanism. The affected subpopulation (red squares) shows metabolic impairment due to the constitutive CYP3A5*3/*3 genotype and predicted poor metabolizer (fPM) status, indicating that critical drug exposure results from different genetic and environmental causes.

Group D (Critical Inhibition) represents a distinct pathophysiological mechanism marked by genetic vulnerability and potent DDIs. This cohort shows normal baseline inflammatory profiles, with low mean CRP levels of 6 mg/L (P9) and 5 mg/L (P18), excluding cytokine-mediated suppression. The metabolic failure in Group D results from a homozygous loss-of-function CYP3A5*3/*3 non-expressor genotype combined with a potent, mechanism-based irreversible CYP3A4 inhibitor. Without functional CYP3A5-mediated compensatory clearance, the DDI fully inactivates the remaining active CYP3A4 catalytic sites. This enzymatic deficiency reduces intrinsic clearance to the lowest levels in the cohort (0.5 L/h in P9 and 0.45 L/h in P18), causing a rapid transition into a critical non-linear accumulation phase, with quetiapine concentrations rising to 1400 ng/mL and 1500 ng/mL by day 4 (Figure 4).

In both Groups C and D, persistent and markedly elevated supratherapeutic concentrations sustained from day 4 through day 8 lead to significant oversaturation of peripheral receptors, substantially increasing the risk of severe, life-threatening adverse drug reactions (ADRs), including corrected QT prolongation, cardiac arrhythmias, and profound respiratory depression. In contrast, the reference cohort (Group A, Subgroups A.1 and A.2) serves as the physiological baseline, exhibiting consistent kinetic predictability by maintaining steady-state concentrations within the lower range of the therapeutic window (96.17 ± 20.35 ng/mL) and achieving normal clearance rates (0.0–2.3 L/h). This stability demonstrates that genotypic predictions are highly reliable in clinical practice when potent systemic modifiers, such as severe inflammation or extensive DDI burdens, are absent.

Genotype-independent phenoconversion may pose a risk not only in psychiatric treatment, when multiple drugs are co-administered, but also in intensive care units, perioperative care, and in the treatment of autoimmune or infectious diseases, where significant cytokine elevations are observed. Therefore, the recognition and management of inflammation-driven phenoconversion are essential across different clinical contexts, underscoring the need for dynamic patient monitoring and regular assessment of the degree of inflammation to guide safe and effective pharmacotherapies.

Linear regression analysis shows a mechanistic link between calculated DDI scores and steady-state quetiapine plasma concentrations at day 4 (r = 0.74; *p* < 0.001). The DDI score is defined on a normalized scale from −1 to +1, where 0 indicates the reference (unaffected) state; negative values indicate metabolic deceleration (inhibition), and positive values indicate metabolic acceleration (induction). This reveals a continuum of metabolic capacity from strong enzyme inhibition to induction. Patients are divided into four distinct metabolic phenotypes. Group A (grey circles) represents stable baseline controls maintaining metabolic equilibrium within the therapeutic range (100–500 ng/mL, shown by dashed lines). Group B/D (red squares) includes CYP3A5*3/*3 non-expressors with severe enzyme inhibition and critical supratherapeutic drug accumulation. Group C (green diamonds) consists of individuals with inflammation-induced phenoconversion, in which negative DDI scores are associated with marked increases in plasma drug levels. Group E (blue triangles) describes enzyme-induced patients with positive DDI scores and subtherapeutic drug exposure. The blue trend line shows that the DDI scoring framework is a robust surrogate for quantifying the net effect of enzyme-mediated metabolic modulation. It provides a precise clinical metric to predict individual quetiapine exposure risks and the clinical severity of phenoconversion.

### 3.4. Genotype-Phenotype Mismatch and Treatment Failure

In contrast, patients exposed to strong enzyme inducers exhibited a distinct metabolic phenotype, indicating that upregulation of CYP expression can result in a fUM state that supersedes the baseline genotype. Patients P10, P16, and P22, all with the CYP3A5*1/*1 (wild-type) genotype, were exposed to potent inducers, including carbamazepine, phenytoin, or chronic smoking (with polycyclic aromatic hydrocarbons (PAH) acting via the aryl hydrocarbon receptor (AhR)). In patient P22, the combination of carbamazepine and tobacco smoke likely led to a marked increase in CYP3A4 and CYP3A5 expression, potentially mediated by activation of the PXR and AhR nuclear receptors. According to established regulatory mechanisms in the literature, this process plausibly involves increased H3K9 acetylation and chromatin remodeling at the CYP3A loci, which facilitates enhanced binding of transcription factors such as the PXR/RXR heterodimer. Consequently, patient P22 exhibited a substantial increase in quetiapine clearance (CL_ind_ 4.3 L/h), with plasma drug concentrations decreasing from 115 ng/mL on day 1 to 26 ng/mL by day 8, a level well below the threshold for therapeutic efficacy. Comparable trends were observed in P10 and P16 (Group E), who also experienced subtherapeutic drug exposure and clinical non-response. These results indicate that induction can override both genotype and baseline pharmacokinetics, rapidly establishing a UM-like phenotype that increases the risk of treatment failure.

**Figure 5:**
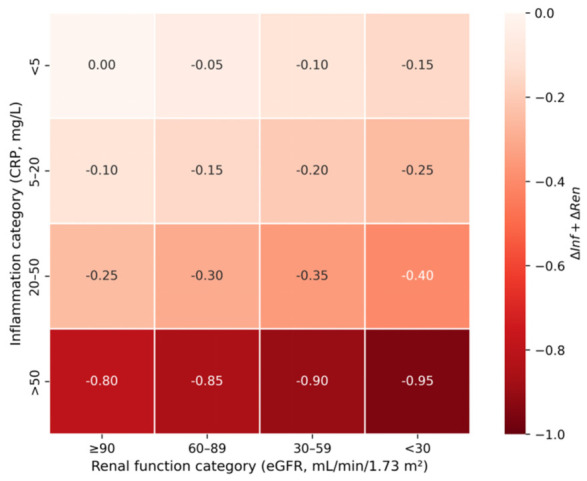
Combined inflammatory and renal modifiers within the P*_act_* framework.

The association between higher P*_act_* scores and preserved metabolic capacity is supported by biological and mechanistic evidence. Systemic inflammation, as indicated by elevated CRP, can reduce CYP3A expression and activity through cytokine-mediated pathways, particularly IL-6 and TNF-α, which inhibit CYP3A transcription and translation. In turn, renal dysfunction, as reflected by reduced eGFR, contributes to phenoconversion by causing the accumulation of uremic toxins and other mediators that inhibit hepatic drug-metabolizing enzymes and cause functional disruption of hepatic homeostasis. The data show that patients with elevated CRP and impaired eGFR had disproportionately reduced functional CYP3A activity, as reflected by lower individual clearance and higher quetiapine concentrations, compared to those with only one risk factor. This suggests a putative synergistic, non-additive effect of inflammation and renal injury on phenoconversion. The combination of increased inflammatory signaling and impaired renal function results in a greater reduction in CYP3A metabolic capacity than either factor alone. This is also observed in lower P*_act_* scores and, independently, in pharmacokinetic parameters, including a steeper decrease in CL_ind_ and increased quetiapine exposure. Because P*_act_* was prespecified based only on genotype, DDI status, CRP, and eGFR environmental modifiers (for genotype), and because CL_ind_ and quetiapine concentrations were excluded from model building, the observed correlations between P*_act_* and these pharmacokinetic outcomes serve as an external, independent test of validity (Figure 5). Thus, the agreement between the higher predicted risk of phenoconversion (lower P*_act_*) and the observed decrease in clearance and increase in exposure empirically supports both the mechanistic plausibility and clinical relevance of the P*_act_* construct.

The heatmap illustrates the additive environmental contribution to CYP3A phenoconversion across predefined clinical strata of systemic inflammation and renal function within the P*_act_* framework. Each cell value represents the combined inflammatory and renal modifier (Δ_Inf_ + Δ_Ren_), calculated from CRP categories and eGFR ranges using the reference-centered Pact model. Higher CRP levels and lower eGFR values produce increasingly negative modifier scores, reflecting greater predicted suppression of CYP3A functional capacity. Final patient-level P*_act_* values are determined by integrating this environmental component with genotype-derived baseline susceptibility and DDI effects. This figure isolates and visualizes the inflammatory–renal dimension of the model.

These findings may have direct implications for clinical practice. Patients with a baseline rapid or extensive metabolizer genotype, or who have been previously exposed to strong inducers, may be particularly susceptible to an abrupt shift to a poor metabolizer phenotype in the presence of systemic inflammation or renal dysfunction. Dynamic monitoring of inflammatory biomarkers, such as CRP and renal biomarkers, combined with pharmacogenetic profiling and systematic assessment of dDDIs, may help identify individuals at risk of phenoconversion-related toxicity or treatment failure at an early stage. The use of composite indices such as P*_act_* in adaptive therapy monitoring may improve dose individualization, reduce toxicity and non-response, and increase the safety of complex polypharmacy.

## 4. Discussion

This study provides a comprehensive, secondary retrospective cohort analysis of the mechanisms underlying phenoconversion in psychiatric polypharmacy. It shows that the metabolic phenotype is dynamic and influenced by multiple factors. Inherited genetic allele patterns and environmental plus physiological factors combine to create the current, changing phenotypic pattern.

This secondary analysis provides mechanistic and pharmacokinetic evidence that CYP3A-mediated drug response in complex psychiatric polypharmacy is governed by a dynamically modifiable phenotype, not genotype alone. In a small but well-characterized cohort, the clinically expressed metabolic state emerges from the convergence of inherited CYP3A5 function, pharmacological crowding, inflammation, renal function, and strong DDIs. Under specific conditions, especially in CYP3A5*3/*3 non-expressors exposed to multi-substrate competition or mechanism-based inhibition, and in inflamed or induced wild-type patients, this interaction causes profound phenoconversion with either toxic overexposure or subtherapeutic underexposure despite apparently favorable or neutral genotypes.

In this study, the controlled internal reference monotherapy subgroup provides a pharmacokinetic baseline with minimal environmental stress, characterized by functional CYP3A5, negligible inflammation, and the absence of relevant DDIs. Here, quetiapine and risperidone concentrations remain within or near the lower therapeutic range, and CL_ind_ values cluster closely around 2.1 L/h, indicating stable and predictable clearance. This baseline allows for quantitative analysis of phenoconversion in other groups and demonstrates that guideline-concordant genotype predictions perform well in the absence of major systemic modifiers.

Compared with the concordant reference group, the CYP3A5*3/*3 subgroup experiencing metabolic crowding (Group B) shows that genetic vulnerability combined with polypharmacy can severely reduce residual clearance. In these individuals, multiple lipophilic CYP3A substrates saturate the remaining CYP3A4 capacity, causing enzymatic crowding, an approximately 75% reduction in CL_ind_, and quetiapine exposures up to 19-fold above the internal baseline and 3.7-fold above the AGNP upper limit. This scenario defines a clinically high-risk phenotype where genetically poor metabolizers cannot rely on CYP3A4 compensation when the catalytic environment is saturated, extending previous *in vitro* and modeling data on competitive CYP3A saturation to a real-world, mechanistically coherent in vivo context. A mechanistically distinct yet equally significant pathway appears in the inflammatory phenoconversion group (Group C). Here, wild-type CYP3A5*1/*1 patients with elevated CRP levels (about 30–55 mg/L) show a strong link between increasing inflammation, reduced CL_ind_, and supratherapeutic quetiapine concentrations, effectively turning genotype-predicted normal metabolizers into a functional poor metabolizer phenotype. This association is seen only within the inflammation-stratified subset, not the overall cohort, supporting a specific cytokine-driven downregulation of CYP3A, consistent with IL-6/TNF-α and PXR/RXR signaling interactions, rather than CRP acting as a nonspecific severity marker. Importantly, these individuals would be classified as “low risk” based on genotype alone, showing that systemic inflammation can fully override pharmacogenetic predictions. Groups D and E illustrate the bidirectional extremes of this dynamic pharmacokinetic system. In Group D, CYP3A5*3/*3 patients receiving strong, mechanism-based inhibitors like ritonavir or cobicistat experience near-complete loss of total CYP3A function (CL_ind_ < 0.5 L/h) and sustained quetiapine concentrations above 1000–1500 ng/mL. This shows that short-term co-prescription can turn guideline-concordant dosing into a toxic regimen. In contrast, Group E shows that potent induction by agents such as carbamazepine, phenytoin, or heavy smoking produces a genotype-independent ultra-rapid metabolizer phenotype. Quetiapine concentrations fall below therapeutic thresholds within days, even in wild-type individuals. Together, these findings indicate that both inhibition and induction can override baseline genotype in opposite directions, causing either severe toxicity or treatment failure, and highlight the need for dynamic, context-sensitive interpretation of pharmacogenetic data.

The heterogeneous phenoconversion mechanisms were integrated into an additive Phenoconversion Model that explicitly separates inherited susceptibility from dynamic environmental modifiers. Modifier scaling was empirically calibrated to keep the genotype–phenotype concordant reference at P*_act_* ≈ 0 and to recapitulate the observed phenotypic hierarchy. Quetiapine concentrations and CL_ind_ were reserved as independent validation endpoints. The strong association between lower P*_act_* scores, reduced clearance, and higher exposure supports the mechanistic plausibility and clinical relevance of this construct. However, its predictive performance requires prospective confirmation. Clinically, these findings suggest that pharmacogenetic information should be interpreted as a dynamic baseline rather than a fixed dosing determinant. It should be continually updated by the current DDI burden, inflammatory state, and renal function. Simple, routinely available markers such as CRP and eGFR, along with structured DDI assessment, could serve as real-time modifiers of genotype-based recommendations. Early, targeted TDM is particularly recommended for gPM or patients with high inflammatory or polypharmacy loads to detect emerging toxicity or underexposure.

This study is exploratory and based on a retrospective cohort. It involves inferred mechanisms and a P*_act_* weighting scheme calibrated in a CYP3A5/quetiapine-dominant context. Therefore, extrapolation to other drugs, populations, and definitive clinical outcomes should be approached with caution. Future multicenter, prospective studies are needed to test P*_act_*-informed adaptive dosing algorithms across CYP3A-sensitive drug classes and to link phenoconversion profiles to molecular readouts and clinical endpoints. Overall, these results support a transition from static, genotype-only dosing toward dynamically adjusted, context-aware precision pharmacotherapy guided by integrated genetic, inflammatory, renal, and DDI information.

## 5. Conclusions

This secondary analysis establishes a new paradigm for understanding and managing phenoconversion in psychiatric polypharmacy, demonstrating that the CYP3A-mediated metabolic phenotype is a dynamic, integrative result of genotype, environmental exposures, and physiological state, rather than a fixed genetic trait. By utilizing a tightly defined genotype-phenotype concordant reference subgroup, within which a monotherapy internal control subgroup provides a clean pharmacokinetic baseline, we were able to quantitatively dissect how specific constellations of risk—CYP3A5*3/*3 non-expression, metabolic congestion, strong inhibition, inflammation, renal failure, and induction—transform real-time metabolic capacity.

Within this framework, the additive P*_act_* model captures phenoconversion as a continuous, clinically interpretable construct that separates baseline susceptibility derived from genotype from environmental and physiological modifiers (DDIs, CRP, eGFR). Within groups, lower P*_act_* scores are consistently associated with reduced intrinsic clearance and disproportionate quetiapine overexposure, including >3-fold deviations from the therapeutic upper limit of AGNP and ≈20-fold increases from intrinsic baseline in extreme cases. In contrast, positive P*_act_* values in inducer-exposed patients are consistent with ultrarapid clearance and subtherapeutic systemic exposure. These concordant patterns indicate that P*_act_* can more accurately quantify real-time metabolic risk than genotype alone, and can unify diverse mechanisms—cytokine-mediated metabolic shutdown, DDI-driven inhibition, and induction-related ultrafast metabolism—into a single, mechanism-informed scale.

However, important limitations also influence these conclusions. The model was developed and calibrated in a small, single-center, quetiapine-dominant cohort, and its current validation is based on pharmacokinetic endpoints rather than hard clinical outcomes. Its performance depends on the completeness and reliability of routinely collected data (DDI profiles, CRP, eGFR), and its behavior in other pharmacological contexts, patient populations, and rare interaction patterns remains uncertain. In summary, our results support P*_act_* and the underlying phenoconversion framework as a mechanistically sound, clinically promising, yet exploratory tool that will encourage a shift from static, genotype-only guidance to dynamically updated, context-aware precision dosing in complex psychiatric care.

## 6. Future Perspectives

Future research should prospectively validate the P*_act_* framework in larger, multicenter cohorts and across additional CYP3A-sensitive drug classes, testing whether P*_act_*-guided adaptive dosing—a combination of PGx, CRP, eGFR, structured DDI assessment, and early TDM—can measurably reduce toxicity and treatment failure compared with current guideline-based care. Parallel mechanistic work linking clinical phenoconversion profiles to cytokine signatures, nuclear receptor activity, and broader multienzyme networks (e.g., CYP2D6, CYP2C19, UGTs, transporters) could refine modifier scaling and support generalization beyond CYP3A5/quetiapine.

From an implementation perspective, embedding P*_act_*-like scores into electronic health record-integrated decision support, with automated extraction of DDIs and key biomarkers, could be a pragmatic next step, but requires standardized data processing and targeted clinician training. Together, these efforts could transform the current proof-of-concept into a scalable, context-aware precision dosing strategy in the high-risk polypharmacy setting.

## Figures and Tables

**Table 1:** Key Factors Contributing to Phenoconversion in Psychiatric Polypharmacy.

Category	Factor (Marker/Component)	Target CYP Enzyme	Effect	Clinical-Laboratory Marker	Reference
Inhibitors	Fluoxetine, Paroxetine	CYP2D6	Strong inhibition	Elevated serum levels	[25,26]
	Ritonavir, Ketoconazole	CYP3A4	Strong inhibition	Phenoconversion: NM → PM	[21,27]
	Risperidone	CYP3A4/3A5	Competitive inhibition	Metabolic crowding	[12,21,27]
Inducers	Carbamazepine, Rifampicin	CYP3A4, 2C9, 2C19	Strong induction	Subtherapeutic levels	[21,27]
	Phenytoin	CYP2C9, 3A4	Induction	Shift from NM to UM	[1,6]
Inflammatory cytokines	IL-6, TNF-α, IL-1β	CYP3A4, 2C19, 1A2	Down-regulation	CRP > 10 mg/L	[6,27]
Clinical markers	Low serum albumin	Protein binding	Increased free fraction	Serum albumin < 35 g/L	[28]
	Renal function (eGFR)	Elimination	Secondary phenoconversion	Decreased eGFR	[2,29,30]
Other factors	Smoking (PAHs)	CYP1A2	Strong induction	Low clozapine/olanzapine levels	[13,29,31,32]
	Chronic alcohol use	CYP2E1	Induction	Increased hepatotoxicity risk	[32,33]

**Table 2:** Classification of CYP3A5-Related Phenoconversion Groups: Genotype, Functional Phenotype, and Primary Drivers.

Groups		*n*	Genotype	Expected Phenotype	Observed Phenotype	Primary Cause of Phenoconversion
Group A	Genotypic Normal Baseline A.1 Monotherapy A.2 No-DDI	6	*1/*1 *1/*3 (GG/GA)	gNM	fNM	Concordant Control: No phenoconversion. Predictive, stable baseline.
Group B	Genotypic Poor with Metabolic Crowding	8	*3/*3 (AA)	gPM	fPM (Collapse)	Substrate-Driven Saturation: Enzyme congestion and competitive saturation due to polypharmacy.
Group C	Genotypic Normal with Inflammatory Phenoconversion	2	*1/*1 (GG)	gNM	fPM (Shift)	Cytokine-Driven Suppression: Inflammatory (CRP > 50 mg/L) phenoconversion with normal genotype.
Group D	Genotypic Poor with Critical Inhibition	2	*3/*3 (AA)	gPM	fPM (Critical)	Mechanism-Based Inhibition: Irreversible enzyme inactivation caused by potent inhibitors (e.g., ritonavir).
Group E	Genotypic Normal with Inducer-Driven Phenoconversion	6	*1/*1 *1/*3 (GG/GA)	gNM	fUM (Shift)	Nuclear Receptor Activation: Transcriptional upregulation caused by xenobiotics (carbamazepine, smoking).

## Data Availability

The retrospective data used in this manuscript are included in the Supplementary File accompanying this publication.

## Supplementary Materials

The following supporting information can be downloaded at: https://www.mdpi.com/article/10.3390/pharmaceutics18070782/s1.

## Abbreviations

BMI	Body Mass Index
CL_ind_	Individual Clearance
CRP	C-Reactive Protein
DDI	Drug–Drug Interaction
eGFR	Estimated Glomerular Filtration Rate
fPM	Functional Poor Metabolizer
fPheno	Functional Phenotype
gIM	Genetically Intermediate Metabolizer
gNM	Genetically Normal Metabolizer
gPM	Genetically Poor Metabolizer
OD	Odds Ratio
PBPK	Physiologically Based Pharmacokinetic Modeling
PGx	Pharmacogenomics
P*_act_*	Phenotypic Activity Score (Dynamic Phenotype)
PCA	Principal Component Analysis
PM	Poor Metabolizer
ROC	Receiver Operating Characteristic
SDDI	Substrate Drug–Drug Interaction (Crowding Index)
TDM	Therapeutic Drug Monitoring
UM	Ultra-Rapid Metabolizer
