# Supplementary material for: Synergistic Effects of Inflammation and Drug Interactions on CYP3A5*3/*3 Phenoconversion in Antipsychotic Metabolism
Source: Pharmaceutics. 2026 Jun 26;18(7):782. doi: 10.3390/pharmaceutics18070782 (PMC13415838; doi:10.3390/pharmaceutics18070782)
Supplement: Supplementary file 1 [file pharmaceutics-18-00782-s001.zip › pharmaceutics-4355113-Supplementary materials S1.pdf]

Patient Identifiers				Concomitant T
Patient ID	CYP3A5 Genotype	Genotypic Phenotype	Dynamic Phenotype	Concomitant Medications
P1	*3/*3	gPM	shift_to_PM	quetiapine;risperidone;clonazepam;atorvastatin
P2	*1/*3	gIM	shift_to_UM	clonazepam;carbamazepine;simvastatin
P3	*1/*1	gNM	stable_NM	quetiapine_only
P4	*3/*3	gPM	shift_to_PM	quetiapine;risperidone;ketoconazole;rosuvastatin
P5	*1/*3	gIM	transient_UM	quetiapine;rifampicin;pravastatin
P6	*3/*3	gPM	persistent_PM	quetiapine;risperidone;clonazepam;atorvastatin
P7	*1/*1	gNM	shift_to_PM_inflam	quetiapine;risperidone;infection;simvastatin
P8	*1/*3	gIM	stable_NM	quetiapine;SSRI;pravastatin
P9	*3/*3	gPM	critical_accumulation	quetiapine;ritonavir;polypharmacy;atorvastatin
P10	*1/*1	gNM	shift_to_UM_smoking	quetiapine;carbamazepine;nicotine;rosuvastatin
P11	*1/*3	gIM	stable_NM	quetiapine;clonazepam
P12	*3/*3	gPM	PM_hypoalbum	quetiapine;risperidone;hypoalbumin;simvastatin
P13	*1/*1	gNM	transient_PM_inf	quetiapine;infection;atorvastatin
P14	*1/*3	gIM	stable_NM	risperidone_only
P15	*3/*3	gPM	severe_PM_DDI	quetiapine;fluoxetine;risperidone;atorvastatin
P16	*1/*1	gNM	shift_to_UM	quetiapine;phenytoin;pravastatin
P17	*1/*3	gIM	shift_to_PM	quetiapine;itraconazole;pravastatin
P18	*3/*3	gPM	critical_accumulation	quetiapine;cobicistat;atorvastatin
P19	*1/*1	gNM	stable_NM	quetiapine;bupropion
P20	*1/*3	gIM	transient_UM	quetiapine;phenobarbital;simvastatin
P21	*3/*3	gPM	PM_due_to_polypharm	quetiapine;erythromycin;atorvastatin
P22	*1/*1	gNM	shift_to_UM	quetiapine;smoking;carbamazepine
P23	*1/*3	gIM	stable_NM	quetiapine;SSRI;rosuvastatin
P24	*3/*3	gPM	severe_PM_DDI	quetiapine;fluconazole;atorvastatin;risperidone

** Pact Score: corrected values per paper Table 2 (Pact = Gbase + Σ(Si × ωi)). Gbase: gNM=0, gIM=−0.5, gPM=−1.0 (normalised scale). Negative Pa*
(highlighted) exceed the upper therapeutic limit.

Therapy	Quetiapine (ng/mL)			Risperidone (ng/mL)			Clona
DDI Mechanism Notes	Day 1	Day 4	Day 8	Day 1	Day 4	Day 8	Day 1
CYP3A crowding + atorva substrate	120	**1850**	320	12	50	48	18
carbamazepine induction; simva substrate	80	45	20	22	10	6	40
monotherapy	90	110	95	0	0	0	0
ketoconazole strong CYP3A inhibitor	60	**920**	640	15	58	52	12
rifampicin induction	130	60	25	30	12	8	35
multi CYP3A substrates	55	420	300	9	40	36	14
high CRP downregulates CYPs	100	**680**	360	20	60	50	25
SSRI mild interaction	70	95	88	18	28	26	20
ritonavir potent CYP3A inhibitor	150	**1400**	980	25	65	60	22
smoking & carbamazepine induction	110	58	30	16	9	5	45
no strong DDI	95	150	130	14	22	20	30
low albumin increases free fraction	85	**760**	520	11	55	50	16
acute infection reduces CYP activity	75	**520**	200	13	48	38	18
monotherapy	0	0	0	20	42	40	0
fluoxetine 2D6 inh + CYP3A crowding	95	**1200**	700	18	62	56	20
phenytoin strong induction	140	55	22	28	11	7	38
itraconazole CYP3A inhibitor	100	980	510	10	56	50	15
cobicistat strong CYP3A inhibitor	130	**1500**	1020	20	68	62	24
bupropion mild induction 2D6	85	120	100	0	0	0	0
phenobarbital induction	125	65	28	26	12	7	36
erythromycin moderate CYP3A inhibition	90	**900**	560	14	54	50	16
smoking + carbamazepine induce clearance	115	60	26	18	10	6	42
SSRI mild + rosuvastatin low interaction	78	110	96	16	30	28	22
fluconazole moderate CYP3A + crowding	98	**1250**	760	17	60	56	21

ct = functional Poor Metabolizer shift; Positive = Ultra-rapid Metabolizer shift. Group A: concordant (Pact=0.00); B: metabolic crowding (−0.60 t

azepam (ng/mL)		Biomarkers & PK						Clinical Factors	
Day 4	Day 8	CRP (mg/L)	Pact Score*	CL_ind (L/h)	Smoking	BMI (kg/m²)	Obesity	Statin	eGFR (mL/min/1.73m²)
55	50	5	-0,74	0,8	No	27	No	Yes	45
0	0	2	0,61	4,5	Yes	24	No	Yes	110
0	0	1	0	2	No	23	No	No	95
48	46	4	-0,72	0,9	No	31	Yes	Yes	50
10	8	3	0,61	4,8	Yes	29	Yes	Yes	120
46	42	8	-0,73	1,1	No	33	Yes	Yes	55
52	48	55	-0,94	0,7	No	28	Yes	Yes	38
30	28	3	0	2,1	Yes	26	No	Yes	100
58	54	6	-0,97	0,5	Yes	36	Yes	Yes	30
12	9	4	0,61	4,2	Yes	30	Yes	Yes	115
42	40	2	0	2	No	25	No	No	98
49	45	7	-0,75	0,9	No	22	No	Yes	40
40	36	28	-0,91	1,4	No	34	Yes	Yes	42
0	0	1	0	2,3	Yes	27	No	No	105
54	50	12	-0,79	0,6	Yes	38	Yes	Yes	28
12	9	3	0,61	4,6	No	24	No	Yes	118
50	46	6	-0,72	0,95	No	32	Yes	Yes	48
62	58	5	-0,97	0,45	Yes	35	Yes	Yes	25
0	0	2	0	2,2	Yes	26	No	No	92
10	8	3	0,61	4,4	Yes	29	Yes	Yes	112
50	46	5	-0,75	0,85	No	31	Yes	Yes	37
11	9	4	0,61	4,3	Yes	29	Yes	No	116
34	32	3	0	2	No	25	No	Yes	99
56	52	9	-0,77	0,7	Yes	37	Yes	Yes	29

o −0.85); C: cytokine-driven (−0.75 to −0.94); D: potent inhibition (−0.90 to −0.98); E: inducer-driven (+0.60 to +0.75). Quetiapine values >500 ng

Group
Group
B
E
A
B
E
B
C
A
D
E
A
B
C
A
B
E
B
D
A
E
B
E
A
B

ɟ/mL

Patient ID	Group	Pact (original file)	Pact (corrected — paper Table 2)	Change
P1	B	**-0,85**	**-0,74**	**0,11**
P2	E	**0,65**	**0,61**	**-0,04**
P3	A	**0,05**	**0**	**-0,05**
P4	B	**-0,7**	**-0,72**	**-0,02**
P5	E	**0,7**	**0,61**	**-0,09**
P6	B	**-0,6**	**-0,73**	**-0,13**
P7	C	**-0,75**	**-0,94**	**-0,19**
P8	A	0	0	0
P9	D	**-0,95**	**-0,97**	**-0,02**
P10	E	**0,6**	**0,61**	**0,01**
P11	A	0	0	0
P12	B	**-0,78**	**-0,75**	**0,03**
P13	C	**-0,55**	**-0,91**	**-0,36**
P14	A	**0,02**	**0**	**-0,02**
P15	B	**-0,9**	**-0,79**	**0,11**
P16	E	**0,68**	**0,61**	**-0,07**
P17	B	**-0,72**	**-0,72**	**0**
P18	D	**-0,98**	**-0,97**	**0,01**
P19	A	**0,08**	**0**	**-0,08**
P20	E	**0,62**	**0,61**	**-0,01**
P21	B	**-0,8**	**-0,75**	**0,05**
P22	E	**0,66**	**0,61**	**-0,05**
P23	A	**0,01**	**0**	**-0,01**
P24	B	**-0,88**	**-0,77**	**0,11**

Note
Corrected to align with paper Table 2 Group B range
Corrected to align with paper Table 2 Group E range
Corrected to align with paper Table 2 Group A range
Corrected to align with paper Table 2 Group B range
Corrected to align with paper Table 2 Group E range
Corrected to align with paper Table 2 Group B range
Corrected to align with paper Table 2 Group C range
Corrected to align with paper Table 2 Group D range
Corrected to align with paper Table 2 Group E range
Corrected to align with paper Table 2 Group B range
Corrected to align with paper Table 2 Group C range
Corrected to align with paper Table 2 Group A range
Corrected to align with paper Table 2 Group B range
Corrected to align with paper Table 2 Group E range
oup C→B (CRP=6 too low for cytokine group; driver is itraconazole inhibition)
Corrected to align with paper Table 2 Group D range
Corrected to align with paper Table 2 Group A range
Corrected to align with paper Table 2 Group E range
Corrected to align with paper Table 2 Group B range
Corrected to align with paper Table 2 Group E range
Corrected to align with paper Table 2 Group A range
Corrected to align with paper Table 2 Group B range

Column	Type	Description
patient_id	Text	Anonymised patient identifier
CYP3A5	Text	CYP3A5 allele genotype
genotype_pheno	Text	Genotype-predicted phenotype
dynamic_phenotype	Text	Observed functional phenotype direction
concomitant_tx	Text	Concomitant medications (INN names)
additional_DDI_notes	Text	DDI mechanism description
quetiapine_day1/4/8	Numeric	Plasma quetiapine concentration
risperidone_day1/4/8	Numeric	Plasma risperidone concentration
clonazepam_day1/4/8	Numeric	Plasma clonazepam concentration
CRP_mg_L	Numeric	C-reactive protein (systemic inflammation)
Pact_Score	Numeric	Phenoconversion Activity Score (CORRECTED)
CL_ind_L_per_h	Numeric	Estimated individual drug clearance
Smoking	Binary	Current smoker status
BMI	Numeric	Body mass index
Obesity_flag	Binary	Obesity (BMI ≥30)
Statin	Binary	Concomitant statin use
eGFR	Numeric	Estimated glomerular filtration rate
Group	Text	Clinical subgroup per paper Table 2

Unit	Standard/Reference
—	PharmVar/CPIC nomenclature
—	PharmVar (*1, *3)
—	CPIC/DPWG terminology
—	Pact model categories
—	WHO INN
—	—
ng/mL	LC-MS/MS; therapeutic range 50–500 ng/mL (AGNP)
ng/mL	LC-MS/MS; typical range 10–60 ng/mL
ng/mL	LC-MS/MS; typical range 10–70 ng/mL
mg/L	Clinical standard; threshold >10 mg/L
Dimensionless (−1 to +1)	Paper Table 2; Pact=Gbase+Σ(Si×ωi)
L/h	Bayesian MAP modelling
Yes/No	Patient history
kg/m^2	WHO standard
Yes/No	WHO definition
Yes/No	Drug list review
$mL/min/1.73m^2$	CKD-EPI equation
A/B/C/D/E	A=concordant; B=crowding; C=cytokine; D=inhibition; E=inducer
