# Supplementary material for: Synergistic Effects of Inflammation and Drug Interactions on CYP3A5*3/*3 Phenoconversion in Antipsychotic Metabolism
Source: Pharmaceutics. 2026 Jun 26;18(7):782. doi: 10.3390/pharmaceutics18070782 (PMC13415838; doi:10.3390/pharmaceutics18070782)
Supplement: Supplementary file 1 [file pharmaceutics-18-00782-s001.zip › pharmaceutics-4355113-Supplementary materials S2 method.pdf]

The most defensible solution is to use genotype as a categorical descriptor not a numerical penalty for gIM and apply baseline penalties only to genetically poor metabolizers (gPM), because they represent established loss of function. This gives :

**Genotypic phenotype**	**Gbase**
gNM	0.00
gIM	0.00
gPM	−0.50

This modeling choice is made to align with our study objective.

Drug-drug interaction modifier

**DDI severity**	**ΔDDI**
None	0.00
Mild inhibition	−0.10
Moderate inhibition	−0.20
Strong inhibition	−0.35
Strong induction	+0.60

Inflammation modifier

**CRP (mg/L)**	**ΔInf**
<5	0.00
5–20	−0.10
20–50	−0.25
>50	−0.80

Renal modifier

**eGFR (mL/min/1.73 m²)**	**ΔRen**

eGFR (mL/min/1.73 m²)	ΔRen
≥90	0.00
60–89	−0.05
30–59	−0.15
<30	−0.25

Final Pact equation: $\mathrm{Pact} = \mathrm{Gbase} + \Delta\mathrm{DDI} + \Delta\mathrm{Inf} + \Delta\mathrm{Ren}$

Methodology section text can be:

"With no validated weights available for CYP3A5-based phenoconversion, the Pact score was formulated based on a reference-based additive model. The baseline score based on genotype and the environmental influences were added using the following equation:

$P_{act} = G_{base} + \Delta_{DDI} + \Delta_{Inf} + \Delta_{Ren}$

where G_{base} is the genotype-based susceptibility, Δ_{DDI} corresponds to the impact of drug-drug interactions, Δ_{Inf} denotes inflammation-induced suppression based on CRP, and Δ_{Ren} corresponds to renal impairment according to eGFR. Modification factors were obtained by clinical cut-offs and pharmacokinetics interactions categories. The modification factors were chosen in an empirical way to keep the concordant genotype-phenotype reference group at P_{act} =0 and reproduce the phenotypic hierarchy as it was observed in patients. The levels of drug concentrations and individual clearances were not part of the Pact determination process but represented the pharmacokinetic endpoint variables separately."
