# Supplementary material for: Synergistic Effects of Inflammation and Drug Interactions on CYP3A5*3/*3 Phenoconversion in Antipsychotic Metabolism
Source: Pharmaceutics. 2026 Jun 26;18(7):782. doi: 10.3390/pharmaceutics18070782 (PMC13415838; doi:10.3390/pharmaceutics18070782)
Supplement: Supplementary file 1 [file pharmaceutics-18-00782-s001.zip › pharmaceutics-4355113-Supplementary materials S2.pdf]

# Supplementary Material

## Individual Patient Data and Pact Score Components

| Patient ID | Group | CYP3A5 Genotype | Gbase | DDI Severity | ΔDDI | CRP (mg/L) | CRP Category | ΔInf | eGFR (mL/min/ 1.73 $m^2$) | eGFR Category | ΔRen | Pact | Quetiapine Day 1 (ng/mL)[1] | Quetiapine Day 4 (ng/mL)[1] | Quetiapine Day 8 (ng/mL)[1] | CLind (L/h) | Primary Driver |
|---|---|---|---|---|---|---|---|---|---|---|---|---|---|---|---|---|---|
| P1 | B | *3/*3 (AA) | -0.50 | Moderate inhibition | -0.20 | 5 | ≤5 | 0.00 | 45 | 30-59 | -0.10 | -0.80 | 120 | 1850 | 320 | 0.80 | Metabolic crowding |
| P2 | E | *1/*1 (GG) | 0.00 | Strong induction | +0.50 | 2 | ≤5 | 0.00 | 110 | ≥90 | 0.00 | +0.50 | 80 | 45 | 20 | 4.50 | Inducer-driven phenoconversion |
| P3 | A | *1/*3 (GA) | 0.00 | None | 0.00 | 1 | ≤5 | 0.00 | 95 | ≥90 | 0.00 | 0.00 | 90 | 110 | 95 | 2.00 | Stable baseline (monotherapy) |
| P4 | B | *3/*3 (AA) | -0.50 | Strong inhibition | -0.25 | 4 | ≤5 | 0.00 | 50 | 30-59 | -0.10 | -0.85 | 60 | 920 | 640 | 0.90 | Metabolic crowding |
| P5 | E | *1/*1 (GG) | 0.00 | Strong induction | +0.50 | 3 | ≤5 | 0.00 | 120 | ≥90 | 0.00 | +0.50 | 130 | 60 | 25 | 4.80 | Inducer-driven phenoconversion |
| P6 | B | *3/*3 (AA) | -0.50 | Moderate inhibition | -0.20 | 8 | 6–20 | -0.10 | 55 | 30-59 | -0.10 | -0.90 | 55 | 420 | 300 | 1.10 | Metabolic crowding |
| P7 | C | *1/*1 (GG) | 0.00 | None | 0.00 | 55 | >50 | -0.80 | 38 | 30-59 | -0.10 | -0.90 | 100 | 680 | 360 | 0.70 | Inflammatory phenoconversion |
| P8 | A | *1/*3 (GA) | 0.00 | None | 0.00 | 3 | ≤5 | 0.00 | 100 | ≥90 | 0.00 | 0.00 | 70 | 95 | 88 | 2.10 | Stable baseline |
| P9 | D | *3/*3 (AA) | -0.50 | Strong inhibition | -0.25 | 6 | 6–20 | -0.10 | 30 | 30-59 | -0.10 | -0.95 | 150 | 1400 | 980 | 0.50 | Critical inhibition (ritonavir) |
| P10 | E | *1/*1 (GG) | 0.00 | Strong induction | +0.50 | 4 | ≤5 | 0.00 | 115 | ≥90 | 0.00 | +0.50 | 110 | 58 | 30 | 4.20 | Inducer-driven phenoconversion |
| P11 | A | *1/*3 (GA) | 0.00 | None | 0.00 | 2 | ≤5 | 0.00 | 98 | ≥90 | 0.00 | 0.00 | 95 | 150 | 130 | 2.00 | Stable baseline |
| P12 | B | *3/*3 (AA) | -0.50 | Moderate inhibition | -0.20 | 7 | 6–20 | -0.10 | 40 | 30-59 | -0.10 | -0.90 | 85 | 760 | 520 | 0.90 | Metabolic crowding |
| P13 | C | *1/*1 (GG) | 0.00 | None | 0.00 | 28 | 20-50 | -0.25 | 42 | 30-59 | -0.10 | -0.35 | 75 | 520 | 200 | 1.40 | Inflammatory phenoconversion |
| P14 | A | *1/*3 (GA) | 0.00 | None | 0.00 | 1 | ≤5 | 0.00 | 105 | ≥90 | 0.00 | 0.00 | N/A | N/A | N/A | 2.30 | Stable baseline (monotherapy) |
| P15 | B | *3/*3 (AA) | -0.50 | Moderate inhibition | -0.20 | 12 | 6–20 | -0.10 | 28 | <30 | -0.15 | -0.95 | 95 | 1200 | 700 | 0.60 | Metabolic crowding |
| P16 | E | *1/*1 (GG) | 0.00 | Strong induction | +0.50 | 3 | ≤5 | 0.00 | 118 | ≥90 | 0.00 | +0.50 | 140 | 55 | 22 | 4.60 | Inducer-driven phenoconversion |

| Patient ID | Group | CYP3A5 Genotype | Gbase | DDI Severity | ΔDDI | CRP (mg/L) | CRP Category | ΔInf | eGFR (mL/min/ 1.73 m²) | eGFR Category | ΔRen | Pact | Quetiapine Day 1 (ng/mL)[1] | Quetiapine Day 4 (ng/mL)[1] | Quetiapine Day 8 (ng/mL)[1] | CLind (L/h) | Primary Driver |
|---|---|---|---|---|---|---|---|---|---|---|---|---|---|---|---|---|---|
| P17 | B | *1/*3 (GA) | 0.00 | Strong inhibition | -0.25 | 6 | 6–20 | -0.10 | 48 | 30-59 | -0.10 | -0.45 | 100 | 980 | 510 | 0.95 | Metabolic crowding |
| P18 | D | *3/*3 (AA) | -0.50 | Strong inhibition | -0.25 | 6 | 6–20 | -0.10 | 25 | <30 | -0.15 | -1.00 | 130 | 1500 | 1020 | 0.45 | Critical inhibition (cobicistat) |
| P19 | A | *1/*3 (GA) | 0.00 | None | 0.00 | 2 | ≤5 | 0.00 | 92 | ≥90 | 0.00 | 0.00 | 85 | 120 | 100 | 2.20 | Stable baseline |
| P20 | E | *1/*3 (GA) | 0.00 | Strong induction | +0.50 | 3 | ≤5 | 0.00 | 112 | ≥90 | 0.00 | +0.50 | 125 | 65 | 28 | 4.40 | Inducer-driven phenoconversion |
| P21 | B | *3/*3 (AA) | -0.50 | Moderate inhibition | -0.20 | 5 | ≤5 | 0.00 | 37 | 30-59 | -0.10 | -0.80 | 90 | 900 | 560 | 0.85 | Metabolic crowding |
| P22 | E | *1/*1 (GG) | 0.00 | Strong induction | +0.50 | 4 | ≤5 | 0.00 | 116 | ≥90 | 0.00 | +0.50 | 115 | 60 | 26 | 4.30 | Inducer-driven phenoconversion |
| P23 | A | *1/*1 (GG) | 0.00 | None | 0.00 | 3 | ≤5 | 0.00 | 99 | ≥90 | 0.00 | 0.00 | 78 | 110 | 96 | 2.00 | Stable baseline |
| P24 | B | *3/*3 (AA) | -0.50 | Moderate inhibition | -0.20 | 9 | 6–20 | -0.10 | 29 | <30 | -0.15 | -0.95 | 98 | 1250 | 760 | 0.70 | Metabolic crowding |

[1] All quetiapine plasma concentration values represent steady-state trough concentrations (Cmin) measured at the end of the dosing interval. Day 1 = pre-dose concentration on first monitoring day; Day 4 = trough at day 4 TDM; Day 8 = trough at day 8 TDM. N/A = data not available for this patient.

Patients with CYP3A5*3/*3 genotype (AA homozygous non-expressors, gPM) are indicated by light grey row shading.
